# Induction of cortical plasticity for reciprocal muscles by paired associative stimulation

**DOI:** 10.1002/brb3.280

**Published:** 2014-10-15

**Authors:** Makoto Suzuki, Hikari Kirimoto, Kazuhiro Sugawara, Makoto Watanabe, Shinobu Shimizu, Ikuyo Ishizaka, Sumio Yamada, Atsuhiko Matsunaga, Michinari Fukuda, Hideaki Onishi

**Affiliations:** 1 Graduate School of Medical Sciences, Kitasato UniversityKanagawa, Japan; 2 School of Allied Health Sciences, Kitasato UniversityKanagawa, Japan; 3 Institute for Human Movement and Medical Sciences, Niigata University of Health and WelfareNiigata, Japan; 4 Department of Rehabilitation Science, Nagoya University Graduate School of MedicineNagoya, Japan

**Keywords:** Human, magnetic stimulation, motor evoked potential, paired associative stimulation, reciprocal inhibition

## Abstract

**Background:**

Paired associative stimulation (PAS) is widely used to induce plasticity in the human motor cortex. Although reciprocal inhibition of antagonist muscles plays a fundamental role in human movements, change in cortical circuits for reciprocal muscles by PAS is unknown.

**Methods:**

We investigated change in cortical plasticity for reciprocal muscles during PAS. PAS consisted of 200 pairs of peripheral electric stimulation of the right median nerve at the wrist at a frequency of 0.25 Hz followed by transcranial magnetic stimulation of the left M1 at the midpoint between the center of gravities of the flexor carpi radialis (FCR) and extensor carpi radialis (ECR) muscles. Measures of motor cortical excitability included resting motor threshold (RMT), GABA_A_-mediated short-interval intracortical inhibition (SICI), and GABA_B_-mediated long-interval intracortical inhibition (LICI).

**Results:**

Motor evoked potential amplitude-conditioned LICI for the FCR muscle was significantly decreased after PAS (*P *=* *0.020), whereas that for the ECR muscle was significantly increased (*P *=* *0.033). Changes in RMT and SICI for the FCR and ECR muscles were not significantly different before and after PAS. Corticospinal excitability for both reciprocal muscles was increased during PAS, but GABA_B_-mediated cortical inhibitory functions for the agonist and antagonist muscles were reciprocally altered after PAS.

**Conclusion:**

These results implied that the cortical excitability for reciprocal muscles including GABA_B_-ergic inhibitory systems within human M1 could be differently altered by PAS.

## Introduction

Reciprocal inhibition functions are of fundamental importance in human movements. Although spinal disynaptic reciprocal inhibition has been shown in previous studies to be produced by Ia-inhibitory interneuron activation through Ia afferent input from a contracting agonist muscle (Tanaka 1974; Day et al. 1984; Kagamihara and Tanaka 1985; Crone et al. 1987; Katz et al. 1991), suppression of antagonist muscle activity is also ensured by the central nervous system. Presumably, this facilitates the passage of Ia inhibitory interneurons from the corticospinal tract or inhibitory volleys that travel from the motor cortex to the antagonist muscle motor neurons (Hoshiyama et al. 1996; Yang et al. 2006; Gerachshenko and Stinear 2007; Giacobbe et al. 2011). In animals, a number of horizontally oriented intrinsic axon collaterals provide inputs to numerous forelimb movement representations (Huntley and Jones 1991). Experimentation in humans (Melgari et al. 2008; Suzuki et al. 2012) has suggested that common cortical site output might deviate to the extensor carpi radialis (ECR) and flexor carpi radialis (FCR) muscles at different “gains” depending upon the final movement that is, performed. Presumably, this is regulated by horizontal cortical projections in the primary motor cortex (M1) that interconnect functionally related neuronal clusters.

Paired associative stimulation (PAS) is an experimental paradigm that is, extensively used to induce plasticity in the human motor cortex. Repetitive pairings of nerve stimulation and cortical transcranial magnetic stimulation (TMS) are timed to ensure near synchronous arrival of the peripheral input and central stimulus at the motor cortex (Ridding and Taylor 2001; Stefan et al. 2002; Kujirai et al. 2006; Castel-Lacanal et al. 2007; Elahi et al. 2012). A long-lasting increase in corticospinal excitability, considered a marker of plasticity within M1, is induced by PAS, and long-term potentiation (LTP)-like processes may also be important (Stefan et al. 2002; Cirillo et al. 2009; Di Lazzaro et al. 2009). A cortical effect of PAS is likely because F waves and H reflexes evoked by electric stimulation are not affected by PAS (Meunier et al. 2007; Mrachacz-Kersting et al. 2007; Roy et al. 2007). In addition, in subjects implanted with an electrode in the cervical epidural space, recordings of corticospinal descending volleys evoked by TMS over the motor cortex indicate that PAS specifically affects the amplitude of later descending I waves, a finding that is, consistent with a cortical origin for aftereffects induced by PAS (Di Lazzaro et al. 2009; Lamy et al. 2010). Several previous human studies have investigated the aftereffects of a PAS intervention on both *γ*-aminobutyric acid (GABA)_A_- and GABA_B_-ergic cortical circuits (Quartarone et al. 2003; Cirillo et al. 2009; Russmann et al. 2009; Elahi et al. 2012). Their results suggested that PAS might induce selective reinforcement of GABA_B_- and not GABA_A_-ergic cortical circuits. In addition, Castel-Lacanal et al. (2007) reported that PAS using motor point stimulation on the ECR and TMS increased the motor evoked potential (MEP) amplitude of the ECR muscle in 16 of 17 subjects and the FCR muscle in five of 17 subjects, but no significant changes in MEP amplitudes were observed before and after PAS for either the ECR or FCR muscles. However, the changes over time in the MEP amplitude for reciprocal muscles during PAS and changes in GABA_A_- and GABA_B_-ergic cortical circuits for reciprocal muscles before and after PAS are unknown.

An interaction between PAS and human movements suggests that neuroplasticity induced by PAS could be related to motor learning in such clinical conditions as Parkinson's disease (Morgante et al. 2006; Ueki et al. 2006), schizophrenia (Daskalakis et al. 2008; Frantseva et al. 2008), and Huntington's disease (Orth et al. 2010). It is of fundamental neurological importance to clearly understand the mechanisms behind motor cortex plasticity, as well as probably being necessary to develop strategies that enhance recovery from brain damage in humans. However, even though reciprocal inhibition is crucial in human movement, the changes induced by PAS in cortical circuits for reciprocal muscles remain unknown. If horizontal cortical projections for reciprocal muscles are present within M1, and PAS helps to reinforce cortical circuit transmission efficiency, PAS may be able to simultaneously change the cortical circuits controlling reciprocal muscles. We therefore investigated changes in cortical plasticity for reciprocal muscles during PAS to clarify the organizational processes induced by PAS for reciprocal inhibition.

## Materials and Methods

### Subjects

We based our sample size on a desired 80% statistical power to detect peak-to-peak MEP amplitudes with a 0.80 effect size (*r*) and two-sided *α* of 5%. Insertion of 1-power (0.80), *α* (0.05), and effect size (0.80) values into the Hulley matrix (Hulley 1988) derived a sample size of 9. Accordingly, we recruited 10 subjects each for the measurement of both short-interval intracortical inhibition (SICI) and long-interval intracortical inhibition (LICI). The subjects comprised 20 healthy, neurologically intact, right-handed volunteers (10 men, 10 women; age, 20–29 (mean ± standard deviation [SD], 21.7 ± 2.2) years). We screened the subjects, all of whom were naïve to the experimental purpose of the study, for potential risk of adverse events during TMS (Wassermann 1998). We obtain the written, informed consent of each subject prior to their participation. No subject took any medications nor had any neurological or psychiatric diseases. We determined handedness with The Edinburgh Handedness Inventory (Oldfield 1971), with a mean laterality quotient of 0.9 ± 0.2 (mean ± SD) points. The experimental procedures were approved by the Ethics Committee of Niigata University of Health and Welfare, and we performed the study in accordance with the Declaration of Helsinki.

### TMS

**Figure 1:**
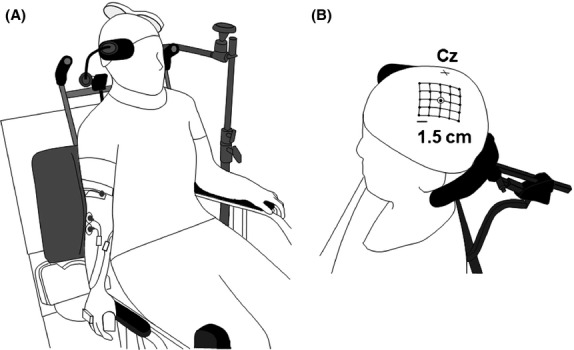
Experimental setup. Change in cortical plasticity for reciprocal muscles during paired associative stimulation was investigated (A). Subjects were seated comfortably in a chair. The right arm hung to the side in a relaxed posture, with the palm and forearm placed on the equipment. Schematic head with a grid showing the stimulated scalp sites (B). Cz represents the intersection of nasion-inion and the interaural lines.

Transcranial magnetic stimulation was delivered as a monophasic current waveform by two Magstim 200 stimulators connected via Y cable (Magstim Co., Ltd., Whitland, Dyfed, U.K.) to the surface of the scalp through a figure-of-eight coil (internal diameter of each wing: 70 mm). After placing a tight-fitting cap over the subject's head, we drew intersecting nasion-inion and interaural lines on the cap with a marker pencil to localize the vertex (Cz) in accordance with the 10–20 International System. To induce a current flow in the left brain from the posterior-lateral to anterior-medial direction, we placed the coil tangentially to the scalp and held the handle pointing backward and sideways, approximately 45° to the midline. As recommended in previous research (Hoshiyama et al. 1996; Hortobágyi et al. 2006; Castel-Lacanal et al. 2007; Giacobbe et al. 2011; Suzuki et al. 2012), we visually detected the optimal coil position to elicit maximum MEPs in each of the FCR and ECR muscles (the “hot spot”) and marked the location with a soft-tipped pen. The subject was comfortably seated in a chair with the right arm allowed to hang to the side in a relaxed posture and the palm and forearm placed on the test equipment. The subject's forearm was held in place by a cushioned support made of particle-foam plastic, with the hand inserted in a hand-piece. The relaxed wrist was held in the equipment in a neutral wrist posture (Fig.1A). The left arm was placed on the armrest and was kept relaxed. We determined the resting motor threshold (RMT) at the hot spot to be the minimum stimulus intensity required to produce a MEP of at least 50 *μ*V in the relaxed FCR and ECR muscles in five of 10 consecutive trials. Throughout this process, we altered stimulus intensity by 1% increments of maximum stimulator output.

### Electromyographic recordings

Before electromyographic (EMG) recording, we reduced the electrical resistance of the skin overlying the FCR and ECR muscles by cleaning it with alcohol. We identified both the FCR and ECR muscle bellies by palpation during manually resisted wrist flexion and extension. Disposable, self-adhesive Ag-AgCl electrodes were placed 2 cm apart over the mid portion of the FCR and ECR muscle bellies in longitudinal alignment with the muscle to record their surface EMG activity. A DL-140 amplifier (4Assisr, Tokyo, Japan) amplified (×100) the EMG signals, which were bandpass filtered at 5–2000 Hz. This data was then digitized at 10 kHz on a PowerLab system (ADInstruments, Colorado Springs, CO) and stored on magnetic media for subsequent retrieval and off-line analysis.

### Motor representational map

The muscle representations were mapped with the aid of a 25-position grid (6 × 6 cm) drawn on the subject's cap, with its center on the respective hot spots of the FCR or ECR muscles (Fig.1B). The hot spot (average value and standard deviation) of the FCR was located at *x* (anteroposterior) = 12.4 ± 8.6 mm and *y* (mediolateral) = 54.3 ± 6.0 mm, and that of the ECR was located at *x *=* *12.0 ± 6.3 mm and *y *=* *52.6 ± 3.1 mm. At each scalp position, the MEPs evoked by five stimulations (interstimulus interval, 5 sec) at 120% of the RMT were recorded in a clockwise spiral course, beginning at the respective hot spot of the FCR or ECR muscle. Map areas corresponded to the stimulated positions. We separately computed the center of gravity (CoG) of each muscle to determine the amplitude-weighted center of the motor representational map (Marconi et al. 2011; Meesen et al. 2011). This was expressed as a bivariate measurement comprising an anteroposterior (*x*) and mediolateral (*y*) coordinate (with Cz reflecting the coordinate origin), according to the following formula:

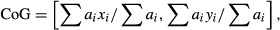
1where *x*_*i*_, *y*_*i*_ are stimulation position coordinates and *a*_*i*_ is amplitude. The CoGs corresponded to the locations of the excitable populations of neurons that project to the target muscles. Usually, optimal coil position for eliciting MEPs is determined according to each muscle's hot spot, but we detected stricter coil positions in this study by calculating CoGs for simultaneously eliciting MEPs from reciprocal muscles. We recorded PAS and cortical excitability at the midpoint between the CoGs of the FCR and ECR muscles because earlier, we found the input-output curves measured midway between the CoGs of both muscles and the CoG of each muscle to be homogeneous (Suzuki et al. 2012). Certain recruitment characteristics of both motor and corticospinal neurons influence the input-output curve (Devanne et al. 1997). Homogeneity of the input-output curves (Suzuki et al. 2012) implies that cortical excitability recordings at the midpoint of CoGs between reciprocal muscles might be an alternative to the separate recording of cortical excitability by stimulating each reciprocal muscle separately. Therefore, we performed PAS by positioning the coil so it would stimulate the reciprocal muscles simultaneously. This allowed for both simultaneous stimulation of reciprocal muscles and successful observation of reciprocal inhibition function.

### PAS

Paired associative stimulation was performed by delivering 200 pairs of peripheral electric stimulation (frequency, 0.25 Hz) to the right median nerve at the wrist. TMS of the left M1 was then performed at the point midway between the CoGs of the FCR and ECR muscles (Ziemann et al. 2004; Delvendahl et al. 2010; Ilic et al. 2011; Kang et al. 2011; Voytovych et al. 2012). Electrical stimulation was applied by an electrical stimulator (Neuropack; Nihon Kohden, Tokyo, Japan) via bipolar electrode with the cathode proximal. After we identified the optimal stimulation site at the wrist, we affixed the electrode and determined the threshold of perception. We applied constant-current square wave pulses of 1000-*μ*sec duration during PAS at an intensity that was three times the perceptual threshold, whereas for TMS, the intensity that produced MEP amplitudes of 130% of the RMT in the FCR muscle was used. The interstimulus interval between the electrical stimulation and TMS was 25 msec. The subjects were constantly reminded to focus their attention on the stimulated hand to ensure that their attention level did not influence the magnitude of the PAS effect (Stefan et al. 2004).

### Cortical excitability recordings

We recorded the peak-to-peak MEP amplitudes evoked by TMS following 25 msec of electrical stimulation during PAS. Measures of motor cortical excitability using TMS included RMT, GABA_A_-mediated SICI, and GABA_B_-mediated LICI (Kujirai et al. 1993) recorded before and after PAS at the point midway between the CoGs of the FCR and ECR muscles. We measured cortical excitability for FCR and ECR muscle, respectively. We also separately measured SICI and LICI to avoid carryover effect and diminishing PAS effect over time. In 10 of the subjects (six men, four women; age, 20–26 [mean ± SD, 21.7 ± 1.6] years), a conditioning stimulus with intensity of 80% of RMT preceded the test stimulus at 120% of RMT for SICI. The interstimulus interval was 3 msec. In the 10 other subjects (four men, six women; age, 20–29 [mean ± SD, 21.7 ± 2.7] years), an intensity of 120% of RMT for both the conditioning and test stimulus and 100 msec as the interstimulus interval for LICI were used. FCR and ECR muscles were relaxed, and RMT was selected as the criterion for the conditioning stimulus because muscle contraction caused a change in agonist-antagonist relation. Moreover, we measured RMT before and after PAS and consistently adjusted conditioning stimulus intensities to be 80% and 120% of RMT for SICI and LICI, respectively, because variation of conditioning stimulus intensity has an influence on cortical excitability (Kujirai et al. 1993; Ziemann et al. 1996; Ilic et al. 2002; Orth et al. 2003). Ten trials of each of unconditioned MEP, SICI, and LICI measurements with a frequency of 0.2 Hz were recorded in random order before and after PAS. Cortical inhibition was calculated as the ratio of conditioned to unconditioned MEP.

### Data analysis

All data are expressed as mean ± standard error of the mean (SEM). Peak-to-peak MEP amplitudes evoked by TMS following 25 msec of electrical stimulation during PAS were averaged at every 10 consecutive stimuli and used for analyses. We compared the difference in MEP amplitudes during PAS between 2 reciprocal muscles (FCR and ECR muscles) and time (time of PAS) with a linear mixed effect model and analyzed differences in RMT, SICI, and LICI before and after PAS with the paired *t*-test. A *P* value of <0.05 was considered statistically significant. PASW Statistics 18 software (IBM, New York, NY) was used for all statistical procedures.

## Results

All subjects completed all experiments. None of the subjects experienced any side effects from TMS during the experiments.

### Motor representational map

**Figure 2:**
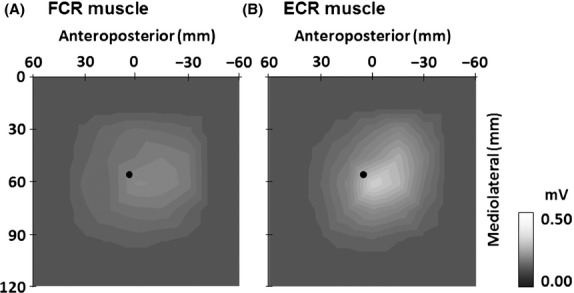
Two-dimensional maps of flexor carpi radialis (FCR) (A) and extensor carpi radialis (ECR) (B) muscles from average data. The color code of each map ranges from white (0 mV) to gray (0.5 mV or over). The vertex (Cz) reflects the coordinate origin. The map areas of FCR and ECR muscles clearly overlapped, although they were spread differently. The center of gravity of the FCR muscle was located at *x* (anteroposterior) = 6.8 ± 2.0 mm and *y* (mediolateral) = 55.7 ± 1.3 mm and that of the ECR muscle at *x *=* *5.6 ± 2.2 mm and *y *=* *55.1 ± 1.4 mm.

The RMTs of the FCR and ECR muscles were 47.1 ± 1.3% and 43.4 ± 1.5% of the maximum stimulator output, respectively. Map areas for the FCR and the ECR muscles are shown in Figure2. The reciprocal muscle areas clearly overlapped, although they were not identical. The CoG of the FCR was located more laterally than that of the ECR in 12 of 20 subjects. The CoG of the FCR was located at *x* (anteroposterior) = 6.8 ± 2.0 mm and *y* (mediolateral) = 55.7 ± 1.3 mm, and that of the ECR was at *x *=* *5.6 ± 2.2 mm and *y *=* *55.1 ± 1.4 mm. The midpoint between the CoGs of the FCR and ECR muscles was located at *x *=* *6.2 ± 2.0 mm and *y *=* *55.4 ± 1.2 mm.

### Change in MEP amplitudes during PAS

**Figure 3:**
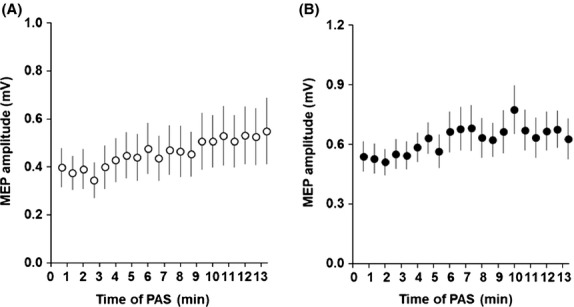
Time-oriented change in peak-to-peak motor evoked potential (MEP) amplitudes of flexor carpi radialis (FCR) (A) and extensor carpi radialis (ECR) (B) muscles during PAS. The time course for peak-to-peak MEP amplitudes during paired associative stimulation (PAS) showed similar patterns.

Time-oriented change in peak-to-peak MEP amplitudes of FCR and ECR muscles evoked by TMS following 25 msec of electrical stimulation during PAS are shown in Figure3 and Table 1. Use of a linear mixed effect model for the analysis of group data revealed that the main effects of time (*F*_19,361_ = 2.710, *P *<* *0.0001) and reciprocal muscles (*F*_1,19_ = 7.37, *P *=* *0.014) were significant, whereas the interaction effect was not significant (*F*_1,19_ = 1.32, *P *=* *0.168). In addition, despite the TMS intensity during PAS (130% RMT) being stronger than TMS intensity for cortical excitability recording before and after PAS (120% RMT), MEP amplitudes at the beginning of PAS were the same or decreased in comparison with those before PAS (before PAS: FCR muscle, 0.39 ± 0.03 mV, ECR muscle, 0.80 ± 0.05 mV; 2 min after the beginning of PAS: FCR muscle, 0.39 ± 0.08 mV, ECR muscle, 0.51 ± 0.07 mV; Tables 1, 2).

**Table 1:** Time-oriented change in peak-to-peak MEP amplitudes during PAS

Time of PAS (min)	MEP amplitudes (mV)	
	FCR muscle	ECR muscle
2	0.39 ± 0.08	0.51 ± 0.07
4	0.43 ± 0.09	0.59 ± 0.07
6	0.48 ± 0.11	0.66 ± 0.10
8	0.46 ± 0.11	0.63 ± 0.10
10	0.51 ± 0.11	0.78 ± 0.12
12	0.53 ± 0.12	0.67 ± 0.10

Values are mean ± standard error of the mean. MEP, motor evoked potential; PAS, paired associative stimulation; FCR, flexor carpi radialis; ECR, extensor carpi radialis.

**Table 2:** MEP amplitudes obtained for the ECR and FCR muscles before and after PAS

	FCR muscle			ECR muscle		
	Before PAS	After PAS	P*	Before PAS	After PAS	P*
RMT (%)	46.7 ± 1.2	46.5 ± 1.4	0.790	44.6 ± 1.0	44.6 ± 1.3	0.949
MEP (mV)	0.39 ± 0.03	0.39 ± 0.02	0.960	0.80 ± 0.05	0.73 ± 0.04	0.009
LICI (conditioned/test MEP)	0.30 ± 0.02	0.23 ± 0.03	0.020	0.22 ± 0.03	0.32 ± 0.05	0.033
SICI (conditioned/test MEP)	0.44 ± 0.04	0.42 ± 0.05	0.595	0.40 ± 0.04	0.40 ± 0.04	0.461

Values are mean ± standard error of the mean. MEP, motor evoked potential; FCR, flexor carpi radialis; ECR, extensor carpi radialis; PAS, paired associative stimulation; RMT, resting motor threshold; LICI, long-interval cortical inhibition; SICI, short-interval cortical inhibition.

* Differences in MEP amplitudes before and after PAS were analyzed by paired *t*-tests.

### Cortical excitability

**Figure 4:**
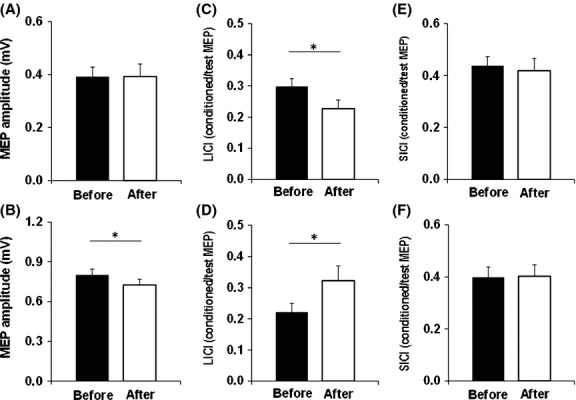
Bar graphs of unconditioned motor evoked potential (MEP) amplitude for flexor carpi radialis (FCR) (A) and extensor carpi radialis (ECR) (B), MEP amplitude-conditioned long-interval cortical inhibition (LICI) for FCR (C) and ECR (D), and MEP amplitude-conditioned short-interval cortical inhibition (SICI) for FCR (E) and ECR (F) before and after paired associative stimulation (PAS). The error bars denote standard error of the mean. MEP amplitude-conditioned LICI for the FCR muscle was significantly decreased after PAS (*P *=* *0.020), whereas that for the ECR muscle was increased (*P *=* *0.033). The change in MEP amplitude for the FCR muscle was not significant (*P *=* *0.960), whereas that for the ECR muscle decreased significantly after PAS (*P *=* *0.009). The changes in SICI for the FCR and ECR muscles were not significant before and after PAS (FCR muscle: *P *=* *0.595, ECR muscle: *P *=* *0.461). *P < 0.05.

Short-interval intracortical inhibition and LICI for the FCR and ECR muscles before and after PAS are shown in Figure4 and Table 2. In the group data analysis using the paired *t*-test, MEP amplitude-conditioned LICI for the FCR muscle was significantly decreased after PAS (*P *=* *0.020), whereas that for the ECR muscle was increased (*P *=* *0.033). The change in MEP amplitude for the FCR muscle was not significant (*P *=* *0.960), whereas that for the ECR muscle decreased significantly after PAS (*P *=* *0.009). The changes in RMT and SICI for the FCR and ECR muscles were not significantly different before and after PAS (RMT, FCR muscle: *P *=* *0.790, ECR muscle: *P *=* *0.950; SICI, FCR muscle: *P *=* *0.595, ECR muscle: *P *=* *0.461).

## Discussion

In the present study, we observed the change in cortical excitability for reciprocal muscles during PAS. The results of this study indicated that (1) the peak-to-peak MEP amplitudes of both agonist (FCR) and antagonist (ECR) muscles evoked by TMS following 25 msec of electrical stimulation were increased during PAS; (2) LICI for agonist (FCR) muscle was enhanced after PAS, whereas that for antagonist (ECR) muscle was disinhibited; and (3) SICI was not changed after PAS. These systematic observations provided evidence that cortical excitability for both reciprocal muscles was affected by PAS, but GABA_B_-mediated cortical inhibitory function for the agonist (FCR) and antagonist (ECR) muscles were reciprocally altered by PAS. To our knowledge, this is the first systematic study to demonstrate the change in cortical excitability for reciprocal muscles during PAS.

Transcranial magnetic stimulation mapping of M1 is based on the idea of stimulating different regions of the brain and measuring the MEPs (Butler and Wolf 2007). The TMS coil position presumably corresponds to the location of the excitable population of neurons, and it projects to the target muscle. TMS maps provide the information on the optimal coil position for activating target muscle at which to obtain the CoG of the area. Huntley and Jones (1991) examined the intrinsic connectivity of M1 for movements of forelimb joints in the monkey and suggested that extensive, horizontally oriented, intrinsic axon collaterals provide inputs to many different forelimb movement representations. In human experimentation, Melgari et al. (2008) and Suzuki et al. (2012) noted that the map areas for FCR and ECR muscles clearly overlap, and the coil position to elicit MEPs of the reciprocal muscles exists at a common site between the CoGs of the FCR and ECR muscles. Therefore, they suggested that the output from the common cortical site might diverge onto FCR and ECR muscles with different “gain” according to the final movement to be performed, presumably regulated by the horizontal cortical projections interconnecting functionally related neuronal clusters within M1 (Melgari et al. 2008; Suzuki et al. 2012). In addition to this possible mechanism, the spread of the electric field might also influence the overlap of TMS maps. The figure-of-eight-shaped coils used this study were more focal, producing maximal current at the intersection of the two round components. One drawback is that the current loops are weakly spread near the intersection of the coil (Rossini et al. 2010). If the neurons that project to the FCR and ECR muscles are located close to each other, FCR and ECR muscles may be simultaneously elicited by spreading of the current. In the present study, the coil position for simultaneously eliciting MEPs from reciprocal muscles was systematically determined. Thereby, PAS was applied with the TMS coil position that simultaneously stimulated reciprocal muscles and peripheral electric stimulation of only the median nerve. This was thought to be the basis for the observation of cortical excitability for reciprocal muscles during PAS. However, we could not determine the mechanism of the common cortical site diverging onto reciprocal muscles and that of the neurons for the reciprocal muscles located close to each other. Although PAS using peripheral electric stimulation of the radial nerve and TMS of the M1 at the CoG of the ECR muscle is technically difficult, further research is needed to investigate the time course of changes in MEPs during PAS using electrical stimulation of radial and median nerves and TMS of M1 at the CoG of both the ECR and FCR muscles.

The first additional new observation in our study was that the time-oriented change in MEP amplitudes evoked by TMS following 25 msec of electrical stimulation during PAS increased on reciprocal muscles. We found a significant increase in peak-to-peak MEP amplitude evoked by TMS following 25 msec of electrical stimulation during conventional PAS (suprathreshold stimulations, 0.25 Hz) interventions. Previous studies (Thickbroom et al. 2006; Rotenberg et al. 2008) demonstrated that 0.2- or 0.25-Hz repetitive TMS without electric stimulation has no plasticity-inducing effect on the human motor cortex. Therefore, the increase of MEP amplitudes for both the FCR and ECR muscles during PAS also may be influenced by PAS. In addition, this increased excitability is thought largely to reflect a change in M1 function because previous studies noted that PAS enhanced responses of later I waves measured with corticospinal descending volleys (Di Lazzaro et al. 2009) and did not change in F waves and H reflexes (Stefan et al. 2000). A part of the projection from the somatosensory cortex to the M1 is organized such that it exhibits high topographical specificity by connecting the homologous somatosensory cortex and M1 (Caria et al. 1997). Ginanneschi et al. (2005) examined the recruitment properties of the corticospinal pathway to intrinsic hand muscles influenced by shoulder joint angle. They suggested that afferent signals registering shoulder position interacted to influence hand muscle recruitment pattern under static conditions. It is, therefore, plausible that peripheral electrical stimulation provides a short-latency input to the M1 via afferents from the somatosensory cortex. In addition to the cause that the output from the common cortical site might diverge onto FCR and ECR muscles (Huntley and Jones 1991; Melgari et al. 2008; Suzuki et al. 2012), there is likely to be afferent divergence from both muscles to both muscle representations. Moreover, MEP amplitudes for reciprocal muscles were decreased at the beginning of PAS in our study. In fact, even though TMS intensity during PAS (130% RMT) was stronger than TMS intensity for cortical excitability recording before and after PAS (120% RMT), MEP amplitudes at the beginning of PAS were the same or decreased in comparison with those before PAS. This is likely because the median nerve stimulation delivered 25 msec before TMS produced MEP inhibition, similar to cholinergic short-latency afferent inhibition (Di Lazzaro et al. 2000; Elahi et al. 2012).

Stefan et al. (2000) examined the topographic specificity of induced plasticity by comparing the effects of PAS on representations of different target muscles. They noted that the MEP amplitudes increased more in the target abductor pollicis brevis (APB) muscle than in the non-target biceps brachii muscle after PAS. Likewise, Ziemann et al. (2004) also demonstrated that MEP amplitude in the APB muscle was increased more than baseline amplitude after PAS. In addition, Castel-Lacanal et al. (2007) performed PAS consisting of an electrical peripheral stimulation of the motor point of the ECR muscle, followed by TMS of the hot spot of the ECR muscle for a period of 30 min. They noted that the MEP amplitude for ECR muscles increased, whereas that for FCR muscles was not significantly changed before and after PAS. In marked contrast to the findings of the Stefan et al. (2000), Ziemann et al. (2004) and Castel-Lacanal et al. (2007), although the time-oriented change in MEP amplitudes evoked by TMS following 25 msec of electrical stimulation during PAS in the present study increased significantly in reciprocal muscles, the MEP amplitudes for both FCR and ECR muscles did not increase before and after PAS. Arányi et al. (1998) noted that MEP facilitation by voluntary contraction varies between muscles. In small hand muscles, the MEP size rises sharply at small forces and levels off at forces above some 5% of the maximum. In more proximal muscles including the FCR muscle, the MEP size increases continuously, with forces increasing by some 25–30%. Arányi et al. (1998) also suggested that these differences are explained by the differences in motor unit recruitment in proximal versus distal muscles. Therefore, one possible explanation for the same MEP amplitude of the FCR muscle before and after PAS in the present study is that the effectiveness of PAS might be related to distal and proximal muscles with different levels of recruitment. In addition, Castel-Lacanal et al. (2007) delivered the electrical peripheral stimulation at the motor point of the ECR muscle and TMS at the hot spot of the ECR muscle at a frequency of 0.1 Hz for a period of 30 min, whereas our study delivered the electrical peripheral stimulation at the median nerve and TMS at the midpoint between the CoGs of the FCR and ECR muscles at a frequency of 0.25 Hz for a period of about 10 min. Another possible explanation is that the M1 plasticity in accordance with topographic specificity was actualized in the Castel-Lacanal et al. (2007) study due to long time-repetitive TMS at the hotspot. However, the optimal TMS frequency and period cited for inducing M1 plasticity for reciprocal muscles is still unclear. Further research is needed to investigate the relation between various PAS protocols and changes in M1 excitability for reciprocal muscles.

We found that PAS intervention failed to modulate GABA_A_-ergic cortical inhibition yet induced an increase in GABA_B_-ergic cortical inhibition for agonist (FCR) muscle, similar to previous studies (Stefan et al. 2000; Morgante et al. 2006; Russmann et al. 2009; Elahi et al. 2012). The second additional new observation in our study was that GABA_B_-mediated LICI for agonist (FCR) muscle was enhanced by PAS, whereas that for antagonist (ECR) muscle was disinhibited. In fact, despite the equal stimulus intensities based on RMT without significant difference before and after PAS, MEP amplitude-conditioned LICIs for the agonist FCR and antagonist ECR muscles were reciprocally changed by PAS. Elahi et al. (2012) found an increase not only in the cortical silent period (CSP) but also in MEP amplitudes after PAS. Because administration of the GABA_B_ receptor agonist baclofen and GABA reuptake inhibitor tiagabine prolonged CSP (Siebner et al. 1998; Werhahn et al. 1999), it is probably mediated through postsynaptic GABA_B_ receptors, similar to LICI. Sanger et al. (2001) suggested that LICI acts primarily through GABA_B_ receptors and inhibits SICI presynaptically, whereas SICI normally activates postsynaptic GABA_A_ receptors and inhibits MEP amplitude. Thus, despite the horizontal cortical projections within M1 for the FCR and ECR muscles, the different effects of PAS interventions on unconditioned MEP amplitude and MEP amplitude-conditioned LICI suggest that the same circuits do not mediate them. However, the precise mechanism of reciprocal change in LICI by PAS in agonist and antagonist muscles is still unclear. TMS over the optimal site for stimulating the FCR muscle was directed at the cortical region that presumably received the maximal peripheral afferent input. Hence, the GABA_B_-ergic cortical plasticity induced by PAS might follow more strict topographical rules than that induced by unconditioned MEP amplitude.

Long-term potentiation-like plasticity is decreased in several neurological and psychiatric disorders with abnormal motor learning, such as Parkinson's disease (Morgante et al. 2006), schizophrenia (Daskalakis et al. 2008), and Huntington's disease (Orth et al. 2010). Ziemann et al. (2004) suggested that synaptic strength of cortical horizontal connections was modified through LTP and long-term depression (LTD). PAS can be used to induce LTP- (PAS at an interstimulus interval of 25 msec) and LTD-like (PAS at an interstimulus interval of 10 msec) effects (Stefan et al. 2000; Rosenkranz et al. 2007). Jung and Ziemann (2009) noted that motor practice depressed subsequent PAS-induced LTP-like plasticity but enhanced PAS-induced LTD-like plasticity. Our results suggest that corticospinal excitability for both reciprocal muscles was increased by PAS; especially, GABA_B_-mediated cortical inhibitory functions for agonist and antagonist muscles were reciprocally altered after PAS. Further research is needed to investigate the relation between cortical excitability and reciprocal function during intervention combining PAS and motor skill training to determine whether PAS induces reciprocal changes in other agonist-antagonist pairs.

In conclusion, we found that cortical plasticity for reciprocal muscles changes during PAS. Our study provided evidence that cortical excitability for both reciprocal muscles was increased during PAS, but GABA_B_-mediated cortical inhibitory functions for the agonist and antagonist muscles were reciprocally altered after PAS. These results implied that cortical excitability for reciprocal muscles including GABA_B_-ergic inhibitory systems within the human M1 could be differently altered by PAS.
